# Duloxetine-Induced Hyponatremia in an Elderly Male Patient with Treatment-Refractory Major Depressive Disorder

**DOI:** 10.1155/2019/4109150

**Published:** 2019-05-13

**Authors:** Ching-Fang Sun, Yeo-Lin Chen, Ying-Hsuan Li, Monika Kumaraswamy, Ying-Chih Lo, Yi-Ting Chen

**Affiliations:** ^1^College of Medicine, China Medical University, Taichung, Taiwan; ^2^Department of Psychiatry & Brain Disease Research Center, China Medical University Hospital, Taichung, Taiwan; ^3^Department of Radiology, China Medical University Hospital, Taichung, Taiwan; ^4^Division of Infectious Diseases, University of California San Diego, La Jolla, CA 92093, USA; ^5^Infectious Diseases Section, VA San Diego Healthcare System, San Diego, CA 92161, USA; ^6^Division of Nephrology, Taichung Veterans General Hospital, Taichung, Taiwan; ^7^Graduate Institute of Biomedical Sciences, China Medical University, Taichung, Taiwan

## Abstract

Several classes of antidepressants can induce syndrome of inappropriate antidiuretic hormone hypersecretion (SIADH), thereby causing hyponatremia. Initial symptoms of hyponatremia include neuropsychiatric and gastrointestinal manifestations can mimic depression, especially in elderly people with multiple somatic complaints. Here we present a case of a 68-year-old man with treatment-refractory depression and general anxiety disorder who developed duloxetine-induced hyponatremia. His symptoms of hyponatremia including unsteady gait, dizziness, nausea, general malaise, and poor appetite subsided after discontinuing the offending medication. Our case illustrates that drug-induced SIADH and potential drug-drug interactions should be considered in elderly patients who develop hyponatremia following the initiation of antidepressants.

## 1. Introduction

Duloxetine and other serotonin-norepinephrine reuptake inhibitors reportedly induce hyponatremia in 5.7% of patients aged 60 or older [1]. Conversely, agomelatine has not been reported to induce hyponatremia [2, 3]. Furthermore, the interactions of drugs with newer antidepressants are insignificant [4]. Large-scale clinical studies evaluating the incidence of hyponatremia in patients utilizing duloxetine are lacking.

The initial symptoms of hyponatremia are primarily neuropsychiatric and gastrointestinal disturbances such as dizziness, clouding of consciousness, psychomotor retardation, confusion, gait impairment, falls, seizures, and nausea/vomiting/diarrhea [5]. Some of them can mimic depression especially in elderly patients with multiple somatic complaints. Herein, we describe a case of hyponatremia secondary to syndrome of inappropriate antidiuretic hormone hypersecretion (SIADH) associated with initiation of duloxetine and potentially exacerbated by agomelatine.

## 2. Case Presentation

A 68-year-old male with a history of treatment-refractory depression, general anxiety disorder, type 2 diabetes mellitus, and benign prostatic hyperplasia presented to our outpatient psychiatric clinic with worsening symptoms of depression including social withdrawal, problems with self-care and inattentiveness. Initially diagnosed with major depressive disorder and general anxiety disorder in 2001, his symptoms initially included depressive mood, anhedonia, psychomotor retardation, hopelessness, and suicidal ideation and were previously well managed with bupropion (150 mg/day) and lorazepam (0.5 mg/day). However, his treatment was ultimately modified to duloxetine (30 mg/day) and agomelatine (25 mg/day) given the signs and symptoms of worsening depression noted on presentation.

**Figure 1:**
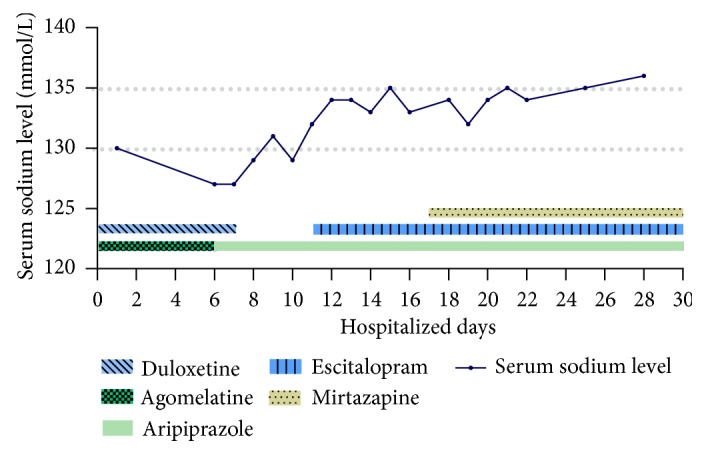
Patient's serum sodium levels and antidepressant treatment regimen during hospitalization. Following discontinuation of agomelatine and duloxetine on the 6^th^ and 7^th^ day of hospitalization the patient's serum sodium level rose from 127 to 135 mmol/L. Ultimately, the patient's depression was well managed with mirtazapine and aripiprazole with tapering of escitalopram.

Following 1 month of treatment with the modified regimen, the patient represented with complaints of unsteady gait, dizziness, nausea, general malaise, poor appetite, constipation, and insomnia. He was subsequently admitted to the hospital for a further workup. Upon interview and examination, he scored 35 on the Hamilton Depression Rating Scale; 91 on the Cognitive Abilities Screening Instrument, Chinese Version, and 35 on the Beck Anxiety Inventory, suggesting severe depression and anxiety without cognitive impairment. Admission laboratory findings were notable for a sodium level of 130 mmol/L (reference range: 135-147 mmol/L) and chloride level of 94 mmol/L (reference range: 98-107 mmol/L) as well as normal renal function: glomerular filtration rate 105 mL/min/1.73 m^2^ (reference range: >90 mL/min/1.73 m^2^); creatinine: 65.416*μ*mol/L (reference range for male: 50-110*μ*mol/L); blood urine nitrogen: 6 mmol/L (reference range: 2.9-7.1 mmol/L), thyroid function: thyroid-stimulating hormone: 2.21 mIU/L (reference range: 0.34-5.60 mIU/L); free thyroxine: 12.87 pmol/L (reference range: 6.94-18.01 pmol/L), and adrenal function: basal serum cortisol level: 563.66 nmol/L (reference range: 170-635 nmol/L). Over the course of the patient's hospitalization, his serum sodium level further decreased to 127 mmol/L and his baseline sodium level prior to the initiation of his latest medication regimen was noted to be 137 mmol/L. A hyponatremia workup was subsequently initiated. He was noted to have an effective serum osmolality of 260 mmol/kg (reference range: 285–300 mmol/kg), urine sodium level of 42 mmol/L (reference: variable), and urine osmolality of 557 mmol/kg (reference range: 300–900 mmol/kg). The patient's symptoms were ultimately attributed to duloxetine-induced hyponatremia associated with SIADH. As a consequence, the duloxetine was discontinued and he was initiated on a high-salt diet, leading to resolution of his hyponatremia (serum sodium: 135 mmol/L) and symptoms including unsteady gait, dizziness, nausea, general malaise, and poor appetite within 8 days (Figure 1). Due to his history and persistent symptoms of depression, he was started on escitalopram titrated from 5 mg/day to 15 mg/day. Ultimately, he was cross-titrated from the selective serotonin reuptake inhibitor (SSRI) escitalopram to the noradrenergic and specific serotonergic antidepressant mirtazapine given concern for the potential development of hyponatremia again while on a SSRI. After 6 weeks of hospitalization, the patient had less psychomotor retardation, dysphoria, and somatic complaints. With improvement in his depression and resolution of his hyponatremia, he was discharged.

## 3. Discussion

Known risk factors for antidepressant-associated hyponatremia are advanced age, female sex, low body weight, low baseline serum sodium, and abnormal potassium level as well as other medical conditions such as heart failure, malignancy, liver disease, adrenal insufficiency, and possibly hypothyroidism [6, 7]. Our patient had several risk factors for hyponatremia including advanced age and low body weight (body mass index: 18.8 kg/m^2^). Generally, hyponatremia is most likely to develop within 2–4 weeks of initiating antidepressants. Thus, trending serum sodium during this time period in patients with known risk factors for hyponatremia is strongly recommended [8].

Unlike those with symptoms of severe acute hyponatremia (serum sodium less than 120 mmol/L) including seizures, coma, and respiratory arrest, our patient presented with only mild symptoms. Unsteady gait is the most prevalent symptom of hyponatremia especially in cases of chronic or mild hyponatremia (serum sodium 130–135 mmol/L) [6]. Other common neuropsychiatric symptoms attributed to hyponatremia include confusion, dizziness/clouding of consciousness, and psychomotor retardation. Gastrointestinal symptoms such as nausea, vomiting, and diarrhea are also commonly observed [4].

Evidence suggests serotonin (5-hydroxytryptamine, 5-HT) and 5-HTergic drugs can induce enhancement of both central 5-HT transmission and antidiuretic hormone (ADH) secretion at the level of the posterior pituitary, resulting in SIADH-associated hyponatremia. Multiple 5-HT receptors (5-HT_2_, 5-HT_4_, and 5-HT_7_ receptors) engage in the regulatory pathway, with 5-HT_2_ receptors being the predominant group [9]. Additionally, dopamine modulates the activity of ADH secretory cells responding to changes in osmolality and stimulates ADH release from the hypothalamus, predominantly via D_2_ receptors [9]. Duloxetine not only inhibits the reuptake of 5-HT and norepinephrine but also increases dopamine, specifically in the prefrontal cortex [10]. Duloxetine can affect multiple neuroendocrine pathways and thereby interfere with ADH secretion.

To objectively evaluate the adverse effect of the drugs administered to our patient, we applied the Naranjo Adverse Drug Reaction Probability Scale (Naranjo Scale) [11]. The score for duloxetine was 4, for agomelatine was 1, for aripiprazole was 0, and for bupropion was −1, suggesting duloxetine and possibly agomelatine were the primary offending medications. Agomelatine is not traditionally associated with hyponatremia but potentially contributed to our patient's SIADH [8, 10]. Agomelatine serves as a melatonin receptor agonist (MT_1_ and MT_2_) and elevates the release of norepinephrine and dopamine due to its 5-HT_2C_ receptor antagonistic effect [12]. Additionally, agomelatine along with duloxetine is metabolized by enzyme CYP2A1 and coadministration of these drugs could prolong each other's half-life [13]. Consistent with both the pharmacokinetic effect and mechanism of action of agomelatine, it may serve as an aggravating factor for the unexpected adverse effect of duloxetine.

A key component of managing drug-induced SIADH is to stop the offending agent. Other mainstays in treating hyponatremia include fluid restriction, a high-salt diet, and intravenous saline as well as the initiation of vaptan drugs in severe cases.

## 4. Conclusion

Hyponatremia is a rare adverse effect of duloxetine; by contrast, agomelatine has not been reported to induce hyponatremia but has a 5-HT_2C_ receptor antagonistic effect and the potential to prolong the half-life of duloxetine. Therefore, caution should be used when prescribing these drugs together given the potential for drug-drug interactions. Patients with risk factors for hyponatremia who are newly initiated on antidepressants should have their sodium levels closely monitored. Once diagnosed with antidepressant-induced SIADH, the potentially offending medications should be stopped immediately, and the patient should undergo a hyponatremia workup.

## Data Availability

The authors confirm that the data supporting the findings of this study are available within the article.

## References

[1] Viramontes T. S., Truong H., Linnebur S. A. (2016). Antidepressant-induced hyponatremia in older adults. *Consultant Pharmacist*.

[2] Taylor D., Paton C., Kapur S. (2015). *The South London and Maudsley NHS Foundation Trust & Oxleas NHS Foundation Trust Prescribing Guidelines in Psychiatry*.

[3] Zajecka J., Schatzberg A., Stahl S., Shah A., Caputo A., Post A. (2010). Efficacy and safety of agomelatine in the treatment of major depressive disorder a multicenter, randomized, double-blind, placebo-controlled trial. *Journal of Clinical Psychopharmacology*.

[4] Lange-Asschenfeldt C., Kojda G., Cordes J. (2013). Epidemiology, symptoms, and treatment characteristics of hyponatremic psychiatric inpatients. *Journal of Clinical Psychopharmacology*.

[5] Spina E., Trifirò G., Caraci F. (2012). Clinically significant drug interactions with newer antidepressants. *CNS Drugs*.

[6] Greenblatt H. K., Greenblatt D. J. (2016). Antidepressant-associated hyponatremia in the elderly. *Journal of Clinical Psychopharmacology*.

[7] Egger C., Muehlbacher M., Nickel M., Geretsegger C., Stuppaeck C. (2006). A review on hyponatremia associated with SSRIs, reboxetine and venlafaxine. *International Journal of Psychiatry in Clinical Practice*.

[8] Lien Y.-H. H. (2018). Antidepressants and hyponatremia. *American Journal of Medicine*.

[9] Iovino M., Guastamacchia E., Giagulli V. A., Licchelli B., Triggiani V. (2012). Vasopressin secretion control: Central neural pathways, neurotransmitters and effects of drugs. *Current Pharmaceutical Design*.

[10] Stahl S. M. (2013). *Stahl's Essential Psychopharmacology: Neuroscientific Basis and Practical Applications*.

[11] Naranjo C. A., Busto U., Sellers E. M. (1981). A method for estimating the probability of adverse drug reactions. *Clinical Pharmacology Therapeutics*.

[12] Valdoxan^®^(agomelatine) [Summary of product characteristics]. Laboratoires Servier ltd, 2009

[13] Lobo E. D., Bergstrom R. F., Reddy S. (2008). In Vitro and In Vivo evaluations of cytochrome P450 1A2 interactions with duloxetine. *Clinical Pharmacokinetics*.

